# Redetermination of clinobaryl­ite, BaBe_2_Si_2_O_7_

**DOI:** 10.1107/S1600536812040457

**Published:** 2012-09-29

**Authors:** Adrien J. Di Domizio, Robert T. Downs, Hexiong Yang

**Affiliations:** a Department of Geosciences, University of Arizona, 1040 E. 4th Street, Tucson, Arizona 85721-0077, USA

## Abstract

Clinobaryl­ite, ideally BaBe_2_Si_2_O_7_ (chemical name barium diberyllium disilicate), is a sorosilicate mineral and dimorphic with baryl­ite. It belongs to a group of compounds characterized by the general formula Ba*M* ^2+^_2_Si_2_O_7_, with *M* ^2+^ = Be, Mg, Fe, Mn, Zn, Co, or Cu, among which the Be-, Fe-, and Cu-members have been found in nature. The crystal structure of clinobaryl­ite has been re-examined in this study based on single-crystal X-ray diffraction data collected from a natural sample from the type locality (Khibiny Massif, Kola Peninsula, Russia). The structure of clinobaryl­ite can be considered as a framework of BeO_4_ and SiO_4_ tetra­hedra, with one of the O atoms coordinated to two Be and one Si, one coordinated to two Si, and two O atoms coordinated to one Si and one Be atom. The BeO_4_ tetra­hedra share corners, forming chains parallel to the *c* axis, which are inter­linked by the Si_2_O_7_ units oriented parallel to the *a* axis. The Ba^2+^ cations (site symmetry *m*..) are in the framework channels and are coordinated by eleven O atoms in form of an irregular polyhedron. The Si—O_br_ (bridging O atom, at site symmetry *m*..) bond length, the Si—O_nbr_ (non-bridging O atoms) bond lengths, and the Si—O—Si angle within the Si_2_O_7_ unit are in marked contrast to the corresponding values determined in the previous study [Krivovichev *et al.* (2004 ▶). *N. Jb. Miner. Mh.* pp. 373–384].

## Related literature

For clinobaryl­ite, see: Chukanov *et al.* (2003 ▶); Rastsvetaeva & Chukanov (2003 ▶); Krivovichev *et al.* (2004 ▶). For clinobaryl­ite-related minerals and compounds, see: Lin *et al.* (1999 ▶); Fleet & Liu (2001 ▶); Kolitsch *et al.* (2009 ▶); Yang *et al.* (2012 ▶). For general information on applications of clinobaryl­ite-related materials, see: Barry (1970 ▶); Robinson & Fang (1977 ▶); Adams & Layland (1996 ▶); Yao *et al.* (1998 ▶); Lu *et al.* (2000 ▶); Yamada *et al.* (2001 ▶); Ohta *et al.* (2004 ▶); Bertaina & Hayn (2006 ▶); Zvyagin (2006 ▶); Zheludev *et al.* (2007 ▶); Yang *et al.* (2012 ▶).

## Experimental

### 

#### Crystal data

BaBe_2_O_7_Si_2_
*M* *_r_* = 323.54
Orthorhombic, 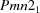

*a* = 11.6491 (5) Å
*b* = 4.9175 (2) Å
*c* = 4.6746 (2) Å
*V* = 267.78 (2) Å^3^
*Z* = 2
Mo *K*α radiation
μ = 7.85 mm^−1^
*T* = 293 K
0.05 × 0.05 × 0.04 mm

#### Data collection

Bruker APEXII CCD area-detector diffractometer
Absorption correction: multi-scan (*SADABS*; Sheldrick, 2005 ▶) *T*_min_ = 0.695, *T*_max_ = 0.744
3929 measured reflections
965 independent reflections
947 reflections with *I* > 2σ(*I*)
*R*_int_ = 0.020

#### Refinement

*R*[*F* ^2^ > 2σ(*F* ^2^)] = 0.011
*wR*(*F* ^2^) = 0.026
*S* = 1.08
965 reflections
60 parameters
1 restraint
Δρ_max_ = 0.47 e Å^−3^
Δρ_min_ = −0.42 e Å^−3^
Absolute structure: Flack (1983 ▶), 399 Friedel pairs
Flack parameter: 0.502 (12)

### 

Data collection: *APEX2* (Bruker, 2004 ▶); cell refinement: *SAINT* (Bruker, 2004 ▶); data reduction: *SAINT*; program(s) used to solve structure: *SHELXS97* (Sheldrick, 2008 ▶); program(s) used to refine structure: *SHELXL97* (Sheldrick, 2008 ▶); molecular graphics: *XtalDraw* (Downs & Hall-Wallace, 2003 ▶); software used to prepare material for publication: *publCIF* (Westrip, 2010 ▶).

## Supplementary Material

Crystal structure: contains datablock(s) I, New_Global_Publ_Block. DOI: 10.1107/S1600536812040457/wm2678sup1.cif

Structure factors: contains datablock(s) I. DOI: 10.1107/S1600536812040457/wm2678Isup2.hkl

Additional supplementary materials:  crystallographic information; 3D view; checkCIF report

## Figures and Tables

**Table:** 

Si—O1	1.6065 (13)
Si—O4	1.616 (2)
Si—O2^i^	1.6315 (14)
Si—O3	1.6566 (10)

**Table:** 

Si^ii^—O3—Si	128.82 (13)

Symmetry codes: (i) 
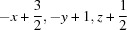
; (ii) 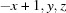.

**Fig. 1.**
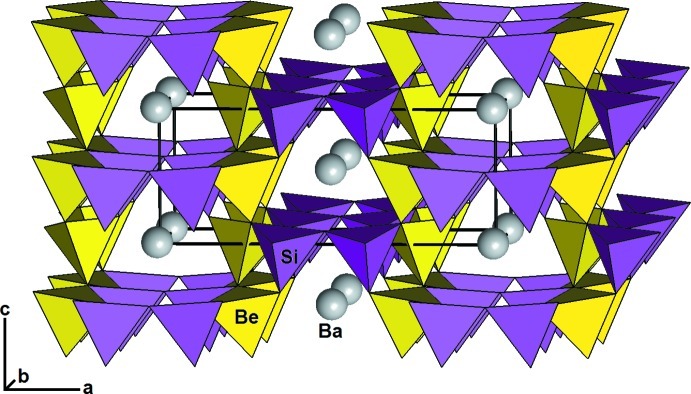
Crystal structure of clinobarylite. The gray spheres represent Ba atoms. The yellow and purple tetrahedra represent BeO4 and SiO4 groups, respectively.

**Fig. 2.**
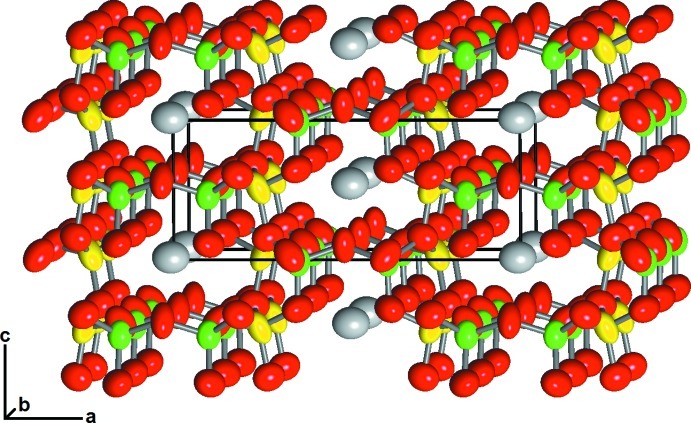
Atoms in clinobarylite with corresponding ellipsoids at the 99.9% probability level. The gray, yellow, green, and red ellipsoids represent Ba, Be, Si, and O atoms, respectively.

**Fig. 3.**
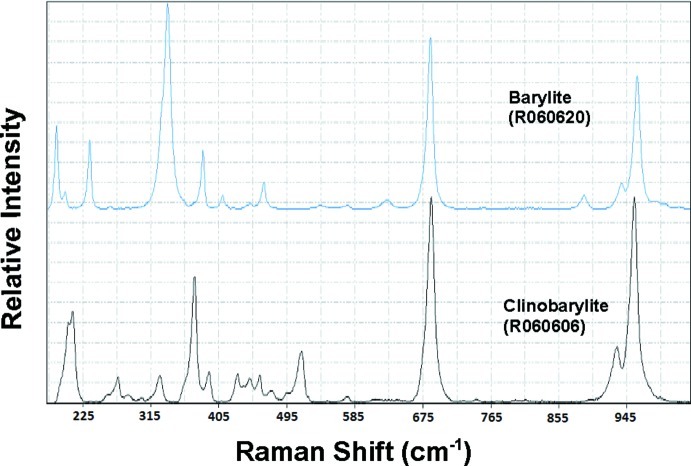
Raman Spectrum of clinobarylite, along with that of barylite for comparison. The spectra are shown with vertical offset for more clarity.

## supplementary crystallographic information

### Comment

Clinobarylite, ideally BaBe_2_Si_2_O_7_, is a sorosilicate mineral and dimorphic with the mineral barylite (Chukanov *et al.*, 2003). It belongs to a group of compounds characterized by the general formula Ba*M*^2+^_2_Si_2_O_7_, with *M*^2+^ = Be, Mg, Fe, Mn, Zn, Co, or Cu (Yang *et al.*, 2012). In addition to the Be-member, the Fe- and Cu-members of this group have also been found in nature, and are known as andrémeyerite and scottyite, respectively. The Ba*M*_2_Si_2_O_7_ compounds have been the subject of numerous investigations for scientific and industrial interests. For example, the materials with *M* = Be, Mg, and Zn are suitable hosts for luminescent activating ions. In particular, Pb^2+^-doped BaBe_2_Si_2_O_7_ is used commercially as an UV-emitting material in moth-killing lamps and (Eu^2+^/Mn^2+^)-doped BaMg_2_Si_2_O_7_ is a deep-red luminescent emitter through effective energy transfers from Eu^2+^ to Mn^2+^ (Barry, 1970; Yao *et al.*, 1998). Moreover, the compounds with *M* = Cu, Co, and Mn are ideal prototypical quasi-one-dimensional quantum spin (*S* = 1/2, 3/2, and 5/2, respectively) Heisenberg antiferromagnets with adjustable superexchange interactions, vital for our understanding of high-*Tc* superconductivity (*e.g.*, Adams & Layland, 1996; Lu *et al.*, 2000; Yamada *et al.*, 2001; Ohta *et al.*, 2004; Bertaina & Hayn, 2006; Zvyagin, 2006; Zheludev *et al.*, 2007).

Clinobarylite was first described by Chukanov *et al.* (2003) as monoclinic with space group *Pm* and unit-cell parameters *a* = 11.618 (3), *b* = 4.904 (1), *c* = 4.655 (1) Å, β = 89.92 (2)°. Its structure was subsequently determined by Rastsvetaeva & Chukanov (2003), yielding a reliability factor *R* = 0.052 with isotropic displacement parameters for all atoms. However, Krivovichev *et al.* (2004) examined the monoclinic structure reported by Rastsvetaeva & Chukanov (2003) and noted that a shift of 0.0088 Å along *b* in all the atomic positions would result in a change of symmetry from monoclinic *Pm* to orthorhombic *Pmn*2_1_. They subsequently collected single-crystal X-ray diffraction data from a new sample, and found that systematic intensity absences were consistent with space group *Pmn*2_1_ and a racemic twinning model (*R*1 = 0.030). Yet, they were unable to obtain positive definite anisotropic displacement parameters for Be and O atoms, and attributed that to the effect of the dominant Ba scattering factor. The resulting geometric parameters, such as bond lengths and angles, matched those obtained from the *Pm* structural model determined by Rastsvetaeva & Chukanov (2003). An examination of the clinobarylite structure reported by Krivovichev *et al.* (2004), nevertheless, reveals a rather peculiar feature: The Si—O_br_ (bridging O atom) distance (1.597 Å) is significantly shorter than the Si—O_nbr_ (non-bridging O atoms) distances (1.619–1.631 Å). This contradicts the previous observations specifically for disilicate compounds (*e.g.*, Lin *et al.*, 1999; Fleet & Liu, 2001; Kolitsch *et al.*, 2009), including all other compounds in the Ba*M*^2+^_2_Si_2_O_7_ group (Yang *et al.*, 2012). The present study was conducted to clarify this controversy.

The crystal structure of clinobarylite is based on a framework of SiO_4_ and BeO_4_ tetrahedra, with one of the O atoms (O2) coordinated to two Be and one Si, one (O3) coordinated to two Si, and two O atoms (O1, O4) coordinated to one Si and one Be. The BeO_4_ tetrahedra share corners to form chains parallel to the *c* axis, which are interlinked by the Si_2_O_7_ units oriented parallel to the *a* axis. The Ba^2+^ cations are situated in the framework channels and are coordinated in form of irregular polyhedra by eleven O atoms if Ba—O distances < 3.4 Å are considered as relevant (Figs. 1, 2). The average Si—O, Be—O, and Ba—O bond lengths in clinobarylite are 1.630, 1.941, and 2.825 Å, respectively. Our study confirmed the space group *Pmn*2_1_ for clinobarylite, as determined by Krivovichev *et al.* (2004), but revealed different bond length from those given by Krivovichev *et al.* (2004): The Si—O_br_ bond length [1.6566 (10) Å] is, in fact, substantially longer than the Si—O_nbr_ bond lengths [1.6065 (13) - 1.6315 (14) Å], in marked contrast to the corresponding values of 1.597 (4) Å, 1.619 (6)–1.631 (7) Å as reported by Krivovichev *et al.* (2004). Moreover, the Si—O_br_—Si angle within the Si_2_O_7_ disilicate unit from our study is 128.82 (13)°, in contrast to 138.5° (Krivovichev *et al.*, 2004).

The Raman spectrum of clinobarylite is plotted in Fig. 3, along with that of barylite (R060620 from the RRUFF Project) for comparison. Evidently, the two Raman spectra are quite similar. In general, they can be divided into four regions. Region 1, between 800 and 1100 cm^-1^, contains bands attributable to the Si—O symmetric and anti-symmetric stretching vibrations (ν_1_ and ν_3_ modes) within the SiO_4_ tetrahedra. Region 2, between 660 and 700 cm^-1^, includes bands resulting from the Si—O_br_—Si bending vibrations within the Si_2_O_7_ tetrahedral dimers. Major bands in region 3, ranging from 420 to 660 cm^-1^, are ascribed to the O—Si—O symmetric and anti-symmetric bending vibrations (ν_2_ and ν_4_ modes) within the SiO_4_ tetrahedra. The bands in region 4, below 420 cm^-1^, are mainly associated with the rotational and translational modes of SiO_4_ tetrahedra, as well as the Be—O interactions and lattice vibrational modes.

One of the noticeable features in Fig. 3 is that the wavenumbers of the bands due to the Si—O_br_—Si bending mode for barylite and clinobarylite are nearly identical (~685 cm^-1^), indicating that the Si—O_br_ bond lengths and the Si—O_br_—Si angles in these two minerals are rather comparable. This is indeed the case. The Si—O_br_ distance and the Si—O_br_—Si angle are 1.657 Å and 128.59°, respectively, in barylite (Robinson & Fang, 1977), and 1.6566 (10) Å and 128.82 (13)° in clinobarylite.

### Experimental

The clinobarylite sample used in this study is from the type locality Yukspor Mountain, Khibiny Massif, Kola Peninsula, Russia, and is in the collection of the RRUFF project (deposition, http://rruff.info/ R060606). The chemical composition of the sample was analyzed with a CAMECA SX50 electron microprobe. The average composition (12 analysis points) is (%_wt_) BaO 47.5 (2), SiO_2_ 37.0 (2), TiO_2_ 0.19 (2), and BeO 15.3 (estimated by the difference from 100%). The empirical chemical formula, calculated on the basis of 7 O atoms, is Ba_1.0_Be_2.0_Si_2.0_O_7_.

The Raman spectrum of clinobarylite was collected from a randomly oriented crystal at 100% power on a Thermo Almega microRaman system, using a solid-state laser with a wavelength of 532 nm, and a thermoelectrically cooled CCD detector. The laser is partially polarized with 4 cm^-1^ resolution and a spot size of 1 µm.

### Refinement

For better comparison with the previous determination of barylite (Krivovichev *et al.*, 2004), the same atom numbering was used, along with the given coordinates as starting parameters for the subsequent refinement. An inversion twin was introduced in the refinement, giving a twin ratio of 0.502 (12):0.498 (12). All atoms were refined with anisotropic displacement parameters. The ideal chemistry, BaBe_2_Si_2_O_7_, was assumed during the final refinement. The highest residual peak (0.47 e^-^/Å^3^) in the difference Fourier maps was located at (0, 0.7651, 0.2479), 0.75 Å from Ba, and the deepest hole (-0.42 e^-^/Å^3^) at (0, 0.3845, 0.0984), 1.75 Å from O4.

### Crystal data

**Table:**

BaBe_2_O_7_Si_2_	*F*(000) = 296
*M**_r_* = 323.54	*D*_x_ = 4.013 Mg m^−^^3^
Orthorhombic, *P**m**n*2_1_	Mo *K*α radiation, λ = 0.71073 Å
Hall symbol: P 2ac -2	Cell parameters from 3074 reflections
*a* = 11.6491 (5) Å	θ = 3.5–32.6°
*b* = 4.9175 (2) Å	µ = 7.85 mm^−^^1^
*c* = 4.6746 (2) Å	*T* = 293 K
*V* = 267.78 (2) Å^3^	Cuboid, colourless
*Z* = 2	0.05 × 0.05 × 0.04 mm

### Data collection

**Table:**

Bruker APEXII CCD area-detector diffractometer	965 independent reflections
Radiation source: fine-focus sealed tube	947 reflections with *I* > 2σ(*I*)
Graphite monochromator	*R*_int_ = 0.020
φ and ω scan	θ_max_ = 32.6°, θ_min_ = 3.5°
Absorption correction: multi-scan (*SADABS*; Sheldrick, 2005)	*h* = −17→16
*T*_min_ = 0.695, *T*_max_ = 0.744	*k* = −7→7
3929 measured reflections	*l* = −6→7

### Refinement

**Table:**

Refinement on *F*^2^	Secondary atom site location: difference Fourier map
Least-squares matrix: full	*w* = 1/[σ^2^(*F*_o_^2^) + (0.0139*P*)^2^] where *P* = (*F*_o_^2^ + 2*F*_c_^2^)/3
*R*[*F*^2^ > 2σ(*F*^2^)] = 0.011	(Δ/σ)_max_ < 0.001
*wR*(*F*^2^) = 0.026	Δρ_max_ = 0.47 e Å^−^^3^
*S* = 1.08	Δρ_min_ = −0.42 e Å^−^^3^
965 reflections	Extinction correction: *SHELXL*, Fc^*^=kFc[1+0.001xFc^2^λ^3^/sin(2θ)]^-1/4^
60 parameters	Extinction coefficient: 0.0160 (9)
1 restraint	Absolute structure: Flack (1983), 399 Friedel pairs
Primary atom site location: structure-invariant direct methods	Flack parameter: 0.502 (12)

### Special details

**Table:**

Geometry. All e.s.d.'s (except the e.s.d. in the dihedral angle between two l.s. planes) are estimated using the full covariance matrix. The cell e.s.d.'s are taken into account individually in the estimation of e.s.d.'s in distances, angles and torsion angles; correlations between e.s.d.'s in cell parameters are only used when they are defined by crystal symmetry. An approximate (isotropic) treatment of cell e.s.d.'s is used for estimating e.s.d.'s involving l.s. planes.
Refinement. Refinement of *F*^2^ against ALL reflections. The weighted *R*-factor *wR* and goodness of fit *S* are based on *F*^2^, conventional *R*-factors *R* are based on *F*, with *F* set to zero for negative *F*^2^. The threshold expression of *F*^2^ > σ(*F*^2^) is used only for calculating *R*-factors(gt) *etc*. and is not relevant to the choice of reflections for refinement. *R*-factors based on *F*^2^ are statistically about twice as large as those based on *F*, and *R*- factors based on ALL data will be even larger.

### Fractional atomic coordinates and isotropic or equivalent isotropic displacement parameters (Å^2^)

**Table:**

	*x*	*y*	*z*	*U*_iso_*/*U*_eq_	
Ba	0.5000	0.20316 (2)	0.5905	0.00932 (5)	
Be	0.75108 (15)	0.1693 (4)	0.0849 (14)	0.0070 (4)	
Si	0.62826 (4)	0.67528 (8)	0.0692 (2)	0.00526 (12)	
O1	0.63895 (10)	0.3560 (2)	0.1367 (3)	0.0073 (3)	
O2	0.77699 (10)	0.1340 (2)	−0.2702 (3)	0.0066 (2)	
O3	0.5000	0.7764 (3)	0.1793 (5)	0.0077 (3)	
O4	0.63272 (12)	0.7295 (2)	−0.2716 (3)	0.0082 (2)	

### Atomic displacement parameters (Å^2^)

**Table:**

	*U*^11^	*U*^22^	*U*^33^	*U*^12^	*U*^13^	*U*^23^
Ba	0.01102 (6)	0.00872 (6)	0.00823 (7)	0.000	0.000	0.00004 (8)
Be	0.0052 (7)	0.0071 (7)	0.0087 (10)	−0.0001 (5)	−0.0020 (17)	0.0017 (18)
Si	0.00460 (16)	0.00420 (14)	0.0070 (3)	−0.00035 (10)	0.0000 (3)	−0.0001 (2)
O1	0.0077 (4)	0.0055 (4)	0.0088 (10)	0.0007 (3)	0.0022 (4)	0.0011 (4)
O2	0.0079 (5)	0.0051 (4)	0.0067 (6)	−0.0008 (4)	0.0007 (4)	0.0003 (4)
O3	0.0045 (7)	0.0082 (7)	0.0103 (9)	0.000	0.000	−0.0024 (6)
O4	0.0075 (6)	0.0109 (5)	0.0064 (7)	−0.0016 (4)	−0.0007 (5)	0.0000 (4)

### Geometric parameters (Å, º)

**Table:**

Ba—O1^i^	2.7721 (13)	Ba—O2^vi^	3.3093 (12)
Ba—O1	2.7721 (13)	Be—O4^vii^	1.591 (3)
Ba—O3^ii^	2.8459 (18)	Be—O1	1.615 (2)
Ba—O4^iii^	2.8689 (13)	Be—O2^viii^	1.670 (4)
Ba—O4^iv^	2.8689 (13)	Be—O2	1.696 (7)
Ba—O4^v^	3.0831 (12)	Si—O1	1.6065 (13)
Ba—O4^vi^	3.0831 (12)	Si—O4	1.616 (2)
Ba—O1^v^	3.1151 (14)	Si—O2^vii^	1.6315 (14)
Ba—O1^vi^	3.1151 (14)	Si—O3	1.6566 (10)
Ba—O2^v^	3.3093 (12)		
			
O4^vii^—Be—O1	116.6 (2)	O1—Si—O2^vii^	114.78 (8)
O4^vii^—Be—O2^viii^	106.0 (3)	O4—Si—O2^vii^	109.70 (8)
O1—Be—O2^viii^	106.8 (2)	O1—Si—O3	107.59 (8)
O4^vii^—Be—O2	107.1 (2)	O4—Si—O3	106.60 (10)
O1—Be—O2	110.4 (3)	O2^vii^—Si—O3	107.14 (8)
O2^viii^—Be—O2	109.90 (19)	Si^i^—O3—Si	128.82 (13)
O1—Si—O4	110.64 (8)		

Symmetry codes: (i) −*x*+1, *y*, *z*; (ii) *x*, *y*−1, *z*; (iii) −*x*+1, *y*−1, *z*+1; (iv) *x*, *y*−1, *z*+1; (v) *x*, *y*, *z*+1; (vi) −*x*+1, *y*, *z*+1; (vii) −*x*+3/2, −*y*+1, *z*+1/2; (viii) −*x*+3/2, −*y*, *z*+1/2.
